# Therapeutic Implications of the Immunoscore in Patients with Colorectal Cancer

**DOI:** 10.3390/cancers13061281

**Published:** 2021-03-13

**Authors:** Carine El Sissy, Amos Kirilovsky, Guy Zeitoun, Florence Marliot, Nacilla Haicheur, Christine Lagorce-Pagès, Jérôme Galon, Franck Pagès

**Affiliations:** 1 INSERM, Laboratory of Integrative Cancer Immunology, Immunology and Cancer department, 75006 Paris, France; carine.elsissy@aphp.fr (C.E.S.); amos.kirilovsky@aphp.fr (A.K.); Florence.marliot@aphp.fr (F.M.); christine.lagorce@aphp.fr (C.L.-P.); jerome.galon@crc.jussieu.fr (J.G.); 2 Equipe Labellisée Ligue Contre le Cancer, 75006 Paris, France; 3 Centre de Recherche des Cordeliers, Université de Paris, 75006 Paris, France; 4 Immunomonitoring Platform, Department of Immunology, Assistance Publique-Hôpitaux de Paris, Georges Pompidou European Hospital, 75015 Paris, France; guy.zeitoun54@icloud.com (G.Z.); nacilla.haicheur@aphp.fr (N.H.); 5 Department of Pathology, Assistance Publique-Hôpitaux de Paris, Georges Pompidou European Hospital, 75015 Paris, France

**Keywords:** Immunoscore, tumor microenvironment, prognosis, predictive response, colorectal cancer, neoadjuvant, treatment, watch and wait, organ preservation, quality of life

## Abstract

**Simple Summary:**

Beyond the TNM-staging system, biomarkers are needed to guide cancer treatments. We provide an overview of the Immunoscore, a standardized immune assay based on quantification by digital pathology of CD3+ and CD8+ cytotoxic T cells in tumor tissues. We discuss the usefulness of the Immunoscore (IS) and biopsies-adapted IS (IS_B_) biomarkers for prediction of clinical outcome and treatment response in colonic and rectal cancers.

**Abstract:**

Four decades were needed to progress from the first demonstration of the independent prognostic value of lymphocytes infiltration in rectal cancers to the first recommendation from the international guidelines for the use of a standardized immune assay, namely the “Immunoscore” (IS), to accurately prognosticate colon cancers beyond the TNM-system. The standardization process included not only the IS conceptualization, development, fine-tuning, and validation by a large international consortium, but also a demonstration of the robustness and reproducibility across the world and testing of international norms and their effects on the IS. This is the first step of a major change of paradigm that now perceives cancer as the result of contradicting driving forces, i.e., the tumor expansion and the immune response, interacting dynamically and influencing the prognosis and the response to therapies. This prompted us to evaluate and evidence the capacity of the tumor immune status, as reflected by the IS, to accurately predict chemotherapy responses in an international, randomized cohort study of colon cancer. Moreover, we developed a derived IS performed on initial diagnostic biopsies (IS_B_) to assess response levels to neoadjuvant therapies. In rectal cancer, IS_B_ was positively correlated with the degree of histologic response to neoadjuvant chemoradiotherapy and identified - alone and even more accurately if combined with clinical data- patients eligible for a noninvasive strategy. Based on these results, we are currently setting up an international cohort for confirmation. The potential role of IS with immunotherapies must be anticipated.

## 1. Introduction

Colorectal cancer (CRC) stands third most in men and second in women with respect to incidence, accounting for 10% of all cancers worldwide. Although its overall incidence is decreasing in many high-income countries, it is increasing in young adults [1]. CRC prognosis relies on histopathological grading, according to the American Joint Committee on Cancer/Union Internationale Contre le Cancer (AJCC/UICC) TNM staging system [2]. However, important differences in clinical outcomes are observed among patients within the same histological tumor stage [3], showing the weaknesses of the TNM classification. Biomarkers are needed to guide the standard of care, such as adjuvant chemotherapy in stage III colon cancer or neoadjuvant chemoradiotherapy (nCRT) in locally advanced rectal cancer (LARC), to better select patients who could benefit from treatment beyond the TNM staging and to predict tumor response to new treatments such as immunotherapies. Many additional tumor cell-based approaches to stratify tumors, like molecular pathways, mutation status, and tumor gene expression [4], have been proposed. However, due to moderate prediction accuracy and reproducibility, these did not translate into the clinical practice, except for the molecular phenotype referred to as MSI (microsatellite instability). All these methods, though, mask intratumor heterogeneity and omit both the tumor microenvironment (TME) and components of the immune system.

Here, we will discuss the usefulness of the recently validated consensus Immunoscore (IS) and biopsies-adapted IS (IS_B_) biomarkers for the prediction of clinical outcome and treatment response in the clinical setting.

## 2. Conceptual Bases and Development of the IS

In the past half-decade, researchers have established that the cancer natural history involves dynamic interactions between the tumor and host defense mechanisms [5]. We now know that a clinically detected cancer evidences the failure of the immune system to eliminate or control alone cancer-cells and to shift to the so-called “immune escape” phase. However, despite this immune-escape shift, immune cells, with the intent to control tumor progression and dissemination, infiltrate tumor glands, surrounding stroma, invasive margin, and the newly formed tertiary lymphoid islets in tumor vicinity [6]. The first report published by Jass et al. in this context demonstrated that a high lymphocyte density evaluated on the histological section in the invasion front of rectal tumors was a prognostic factor independent of the TNM classification [7]. The level of tumor immune infiltration made it possible to specify patients’ prognosis beyond the classical criteria for tumor extension. This seminal observation was thereafter confirmed in many solid tumors and favored by the development of CD recognition-based methods allowing identifying and enumerating lymphocyte subpopulations at the tumor site [4]. However, the immune infiltration and orientation (i.e., type, functional orientation, density, and location of immune cells) were found heterogeneous within tumors. We, therefore, hypothesized that considering each tumor region, we could provide additional information on pathophysiological and possible prognostic levels.

**Figure 1:**
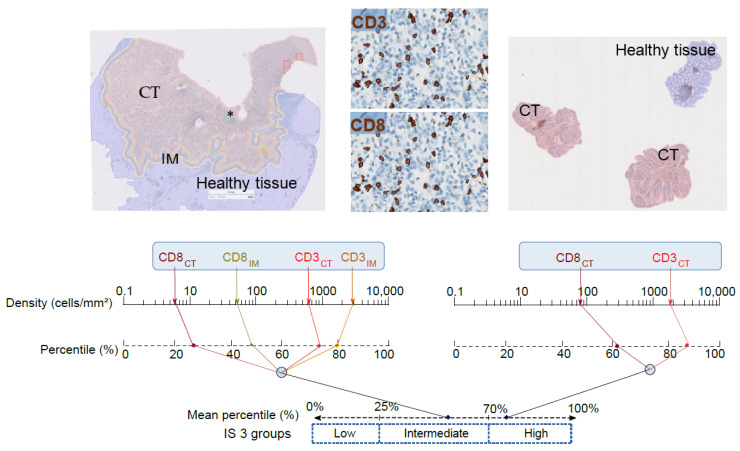
Immunoscore (IS) determination. Top left: Automatic detection of tumor (CT) (pink), invasive margin (IM) (yellow), and healthy tissue (blue) of colon cancer by digital pathology software (IS analyzer, HalioDx; Developer XD Definiens). * Necrosis areas were removed from the analysis. Top right: Representative image of rectal biopsies with tumor region (pink) and normal tissue or dysplasia excluded from the analysis (blue). Top middle: CD3^+^ and CD8^+^ T cells numbers automatic detection. Bottom: Chart illustrating the IS colon (left) and IS_B_ (right) calculation methods: Densities of CD3^+^ and CD8^+^ T cells in the tumor and invasive margin were converted into percentile values in colon cancer, while in rectal biopsies, densities were measured in the CT region only. The mean percentile of the densities was then calculated to generate IS percentile value, where IS_B_ low, IS_B_ intermediate, and IS_B_ high subgroups are reflected by 0–25%, >25–70%, and >70–100% percentile, respectively.

We thus measured the densities of immune cells in tumor core (CT) and invasive margin (IM) of CRC and repeatedly found that the immune ‘contexture’ (i.e., type, functional orientation, density, and location of immune cells) [8,9] was the strongest prognostic factor for tumor dissemination and survival of patients with CRC, whatever their stages. Therefore, we derived a test named IS, based on quantification by digital pathology of CD3+ and CD8+ cytotoxic T cells, in the CT and IM regions in order to transfer this discovery to the clinic (Figure 1). An international validation study confirmed the robustness and the prognostic value of the IS and, what is more, its superiority compared to the AJCC/UICC-TNM staging system [10]. Still, the IS analytical performance was mandatory to prove its capacity to contribute to the worldwide routine practice of prognosis prediction. High-performance analytics confirmed the reliability, reproductivity, and robustness of the IS [11]. Optical and automatic counts of CD3+ or CD8+ cells were strongly correlated (r = 0.94, *p* < 0.001 and r = 0.92, *p* < 0.001, respectively). The CD3 and CD8 staining intensities were not altered by the age of the tumor block over a period of 30 years. Neither the position of tested tissue sections within a tumor block nor the selection of the tissue blocks affected the IS performance. The IS reproducibility was not affected by multiple variables (e.g., antibody lots, DAB revelation kits, immunohistochemistry automates, and operators). The IS inter-assay repeatability inter-laboratory reproducibility between two testing centers reached 100% and 93%, respectively. As a result of this long process, the IS is the first biomarker recommended by academic institutions quantifying the tumor immune infiltrate for a prognostic purpose (the ESMO guidelines 2020 [12] and the 5^th^ edition of WHO Digestive System Tumors [13]).

This lengthy process, in our opinion, is just the first step of a major change of paradigm that no longer considers cancer through the unique scope of cancer cells. Instead, cancer must be perceived as the result of contradicting driving forces interacting dynamically that include not only the cancer cells and the immune environment but also medical treatments (e.g., surgery, chemotherapy, radiation therapy, physical therapy, immunotherapy). This prompted us to evaluate the capacity of the IS to predict the response to specific treatments or therapeutic strategies in colorectal cancer.

## 3. IS and Adjuvant Chemotherapies in Colon Cancer (CC)

The current standard of care for stage III CC is adjuvant therapy with fluoropyrimidine and oxaliplatin [12]. The question of whether the tumor immune status might further determine the extent of response to chemotherapy was recently addressed by two studies of stage III CC.

The pre-defined consensus IS was evaluated in 763 patients with AJCC/UICC-TNM stage III CC derived from the international Immunoscore study [14]. Interestingly, only patients with an Intermediate (Int) or High-Immunoscore responded to chemotherapy and had prolonged survival compared to patients not receiving chemotherapy (HR, 0.42; 95% CI, 0.25–0.71; *P* = 0.0011). In contrast, patients with a low Immunoscore did not significantly benefit from chemotherapy treatment neither in high-risk (*P* = 0.12) nor in low-risk (*P* = 0.17) group. Confirmation of this result by a randomized study could help to select patients who will benefit from an adjuvant treatment and avoid harmful chemotherapy in other settings.

**Figure 2:**
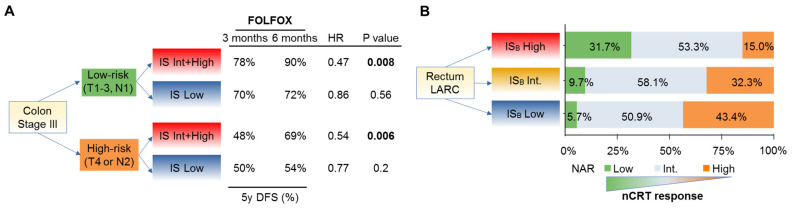
Treatment response and survival (TTR) according to the Immunoscore (IS) categories. (**A**) Impact of the IS on survival (disease-free survival) in stage III colon cancer patients from the IDEA randomized clinical trial categorized by risk subgroups, low and high, according to FOLFOX adjuvant chemotherapy duration. (**B**) The frequency of patients in each IS_B_ groups, according to the neoadjuvant rectal (NAR) score in locally advanced rectal cancer patients.

We also investigated the ability of the IS to predict response to adjuvant chemotherapy in the Immunoscore-International Duration Evaluation of Adjuvant Chemotherapy (IDEA) France phase III trial (*n* = 1062 patients, conducted in collaboration with PRODIGE, a digestive oncology French intergroup (GERCOR, FFCD, UNICANCER)) [15], which aimed to evaluate the noninferiority of three versus six months of adjuvant therapy with either FOLFOX or CAPOX in patients with resected stage III CC [16]. For FOLFOX treated patients (91.6% of the cohort), a statistically significant interaction was observed for the predictive value of the IS for treatment duration (three vs. six months) in terms of DFS. Intermediate (Int.) or high Immunoscore significantly predicted benefit of six months treatment (HR = 0.53; 95% CI, 0.37–0.75; Log-rank *P* = 0.0004), including clinical low-risk (T_1–3_ N_1_) and high-risk (T_4_ or N_2_) stage III CC (all *P* < 0.001). Conversely, patients with low Immunoscore (46.4%) did not derive significant benefits from the six-month FOLFOX versus three-month. These patients appeared to be doubly penalized by an increased risk of recurrence and the lack of benefits from a longer duration of treatment (Figure 2A).

It has been shown that 5-fluorouracil may partially deplete or transiently inactivate inhibitory immune cells [17], while oxaliplatin elicits bona fide immunogenic cell death [18]. Chemotherapy activity could thus, in part, be mediated by an anti-tumor immune response that might eliminate disseminated tumor cells after cancer resection. Intratumoral immune infiltration before treatment might reflect this capacity of the immune system. Therefore, the IS, beyond prognosis, has a predictive value for chemotherapy treatment response. This prompted us to test it in rectal cancer (i.e., LARC) treated with nCRT (nCRT).

## 4. The IS and IS_B_ in Rectal Cancer

Rectal cancer is a major health issue, being the eighth most common cancer worldwide [19]. Combined-modality management strategies, i.e., nCRT, and surgery with total mesorectal excision, have improved outcomes by decreasing local recurrence and distant metastasis. However, there is a necessity to improve long-term quality of life (QoL) in patients with mid and low LARC (T3-T4, N0 or Tx, N1–2, M0) regarding digestive, urinary, and sexual dysfunctions secondary to nCRT and radical surgery.

A complete pathologic response post-nCRT (i.e., no residual tumor) is obtained in about 20% of LARC patients, a state associated with a low-risk of distant and local relapses [20]. Such observation has prompted to propose a preserving strategy, the so-called “Watch and Wait” strategy, to avoid radical surgery, postoperative complications, and short-term poor QoL [21]. There is a crucial need for biomarkers to (i) predict the quality of response to nCRT, to (ii) select patients unlikely to respond to nCRT and thus justifying an nCRT intensification or drug repositioning, and to (iii) better select those with clinical complete response eligible to noninvasive strategy. Although several molecular biomarkers (in tumor tissues or blood) have been proposed as predictive of response to nCRT, none of these have reached the clinic [22].

A derivation of the IS performed in initial diagnostic biopsies (IS_B_) before nCRT has the advantage of evaluating the effect of the initial immune infiltrate (i.e., CD3^+^ and CD8^+^ T cells in the tumor) on both response to nCRT and clinical outcome (Figure 1). In addition, given that nCRT induces architectural and histological changes, post-nCRT surgical specimen cannot be assessed by the classical IS. IS_B_ was tested in a multicentric cohort of 249 patients with LARC treated with nCRT followed by radical surgery [23]. The IS_B_ levels correlated with the degree of histologic response to nCRT according to: (i) The NAR score [24] (Figure 2B), (ii) the Dworak classification [25], and (iii) the ypTNM staging, i.e., the post-surgical pathologic examination (all *P* < 0.001). As an example, patients with IS_B_ high were not found in the Dworak 0 non-responder group (no histologic response to nCRT) and the majority of patient with IS_B_ low (80%–90%) did not respond well to nCRT (no downstaging, Dworak 0, 1, or 2, or NAR low, or Int. categories).

Importantly, IS_B_ combined with post-nT imaging increased the accuracy of histologic good responders (ypTNM 0-I) prediction. This information is of particular importance since imaging is the gold standard in clinical practice to select patients eligible for the Watch and Wait strategy. The clinical utility of the composite biomarker (imaging + IS_B_) was tested within a cohort of “Watch and Wait patients” (*n* = 73) with post-nCRT clinical complete response (ycTNM 0). There was no evidence of relapse during the follow-up period in patients with IS_B_ high. These results suggest that IS_B_ could be a novel biomarker that might be used in the clinic for better selection of patients eligible for the Watch and Wait strategy. We are currently validating this IS_B_ application in a large, multicentric, international cohort of patients with LARC.

Moreover, results observed in studies of patients with stage III CC treated by chemotherapy suggest that IS_B_ can be used to predict response to other standards of care or newer treatments, such as adjuvant chemotherapy [26], total neoadjuvant therapy [26], or high dose CRT [27,28], in LARC patients.

## 5. Immunoscore and Immunotherapy

The impressive results of cancer immunotherapies and checkpoint inhibitors in the past few years have revolutionized the field of oncology. The clinical application of monoclonal antibodies against CTLA-4, PD-L1, and PD-1 molecules resulted in regulatory approvals in a growing number of indications since 2011 [29]. They have been shown to be effective in more than 30 different cancer indications, including microsatellite unstable-high (MSI-H) or deficient mismatch repair (dMMR) colorectal cancer subtypes with distant metastases.

In non-randomized phase II studies, complete/partial response or stable disease was reached in 50% to 90% of dMMR/MSI-H metastatic CRC (mCRC) [30]. Pembrolizumab (anti-PD-1 mAb) is under evaluation in first-line phase III randomized controlled trial (Keynote-177 study, NCT02563002) evaluating the efficacy and safety of anti-PD-1 mAb versus standard of care chemotherapy in MSI/dMMR mCRC. At the second interim analysis, pembrolizumab alone led to significantly longer progression-free survival than chemotherapy with fewer treatment-related adverse events. The estimated percentages of patients alive and progression-free at 24 months were 48.3% and 18.6% in the pembrolizumab and chemotherapy groups, respectively [31].

Biomarkers are needed to select patients who will benefit the most from immune checkpoint inhibitor therapies or to define which type of treatment is more precise or personalized. IS could be one of such markers. Indeed, in a case report by Chakrabarti et al., 12 dMMR mCRC patients treated with pembrolizumab and CD3^+^ and CD8^+^ T cell densities were evaluated in CT and IM [32]. All median T cell densities were higher in responders versus non-responders and in those with a longer duration of disease control. This result is consistent with the use of IS as a biomarker in dMMR mCRC. Moreover, other clinical trials of different immunotherapies are ongoing in dMMR mCRC and biomarkers as the IS will also be required to predict the performance of such treatments [30,33].

However, only 5% of patients with mCRC are dMMR/MSI-H. In the setting of proficient mismatch repair (pMMR) or microsatellite stability (MSS), no immunotherapy has been approved due to a lack of sufficient clinical benefits. mCRC pMMR are largely unresponsive to monotherapy with ICI. Nevertheless, many ongoing clinical trials with different types and combinations of immunotherapies (cancer vaccines, bi-specific, antibodies, mAB against LAG3, TIGIT, anti-TGFβ…) are tested to break the tumor resistance [34]. These will also require precise and reliable biomarkers to predict patient responses to these specific treatments. In several tumor types, a high T cell infiltration has been shown to increase the probability of response to immune checkpoint inhibitor therapies.

Recent studies have demonstrated a very strong pathological response to neoadjuvant immune checkpoint inhibitor therapy in early-stage melanoma, lung cancer, and bladder cancer. Therefore, it was administered in 40 CRC dMMR and pMMR patients (stages I–III) [35]. Strikingly, a major pathological response was observed in all dMMR tumors. Those with pMMR tumors (*n* = 15) showed three major pathological responses and seven partial responses. Interestingly, CD3^+^ and CD8^+^ T cells densities seemed lower in pretreatment biopsies of non-responder patients [35]. Neoadjuvant immunotherapy in early-stage CRC patients might become a new standard of care in those with dMMR tumors and possibly in a subgroup of those with pMMR tumors, possibly improving their surgical outcomes. IS_B_ might guide the selection of patients who are likely to benefit from neoadjuvant immunotherapy in particular those with pMMR tumors.

## 6. Conclusions

The IS has demonstrated its prognostic and predictive values in CC and is now integrated into the guideline recommendations for patients. It might help to select patients who are likely to benefit the most from a longer adjuvant chemotherapy in CC stage III and also, while adapted to biopsies, to identify rectal cancer patients who might respond to neoadjuvant therapy and to be eligible to noninvasive strategies. Many immunotherapies are currently tested with promising results. The IS might constitute a powerful tool to assess response to these immunotherapies. Thereby, the IS could have a strong clinical utility in CRC patients to tailor the gold standards and novel treatments.
